# Development of a Panel of Genotyping-in-Thousands by Sequencing in *Capsicum*

**DOI:** 10.3389/fpls.2021.769473

**Published:** 2021-10-26

**Authors:** Jinkwan Jo, Youngin Kim, Geon Woo Kim, Jin-Kyung Kwon, Byoung-Cheorl Kang

**Affiliations:** Department of Agriculture, Forestry and Bioresources, College of Agriculture and Life Sciences, Research Institute of Agriculture and Life Sciences, Plant Genomics and Breeding Institute, Seoul National University, Seoul, South Korea

**Keywords:** amplicon sequencing, GBS, marker assisted breeding, *Capsicum* spp., pepper, GT-seq, population structure, PCA

## Abstract

Genotyping by sequencing (GBS) enables genotyping of multiple loci at low cost. However, the single nucleotide polymorphisms (SNPs) revealed by GBS tend to be randomly distributed between individuals, limiting their direct comparisons without applying the various filter options to obtain a comparable dataset of SNPs. Here, we developed a panel of a multiplex targeted sequencing method, genotyping-in-thousands by sequencing (GT-seq), to genotype SNPs in *Capsicum* spp. Previously developed Fluidigm^®^ SNP markers were converted to GT-seq markers and combined with new GT-seq markers developed using SNP information obtained through GBS. We then optimized multiplex PCR conditions: we obtained the highest genotyping rate when the first PCR consisted of 25 cycles. In addition, we determined that 101 primer pairs performed best when amplifying target sequences of 79 bp. We minimized interference of multiplex PCR by primer dimer formation using the PrimerPooler program. Using our GT-seq pipeline on Illumina Miseq and Nextseq platforms, we genotyped up to 1,500 (Miseq) and 1,300 (Nextseq) samples for the optimum panel size of 100 loci. To allow the genotyping of *Capsicum* species, we designed 332 informative GT-seq markers from Fluidigm SNP markers and GBS-derived SNPs. This study illustrates the first application of GT-seq in crop plants. The GT-seq marker set developed here will be a useful tool for molecular breeding of peppers in the future.

## Introduction

Backcross breeding introduces a desired trait from a donor parent to an elite line lacking this trait, followed by multiple rounds of backcrosses to restore as much of the genetic background of the elite line in the new germplasm (Allard, 1999). In traditional backcross breeding, backcrossing is repeated for at least five generations to sufficiently dilute the genetic information of the donor parent, outside of the trait of interest. After each backcross, the progeny needs to set seeds to allow phenotyping-based selection, making traditional backcross breeding a lengthy process. Marker-assisted backcrossing (MABC) aims to speed up selection by combining foreground selection to select useful traits and background selection to evaluate the residual genetic contribution of the donor parent to the elite line genome (Charcosset, 1997). MABC, therefore, makes it possible to develop elite lines harboring novel useful traits in only two backcross generations (Visscher et al., 1996; Frisch et al., 1999; Collard and Mackill, 2008). For example, to facilitate MABC, the 412 Fluidigm single nucleotide polymorphism (SNP) markers evenly distributed on each chromosome were developed using transcriptome sequencing data in pepper (*Capsicum annuum*) (Kang et al., 2014). By utilizing the MABC marker set, individuals with 96% of the recurrent parental genome could be obtained in the second backcross (Jeong et al., 2015).

Genotyping hundreds of loci in hundreds of samples is a prerequisite when examining the genetic background of each individual by MABC. Electrophoresis-based molecular markers are time-consuming and labor-intensive to use when analyzing the genotypes of numerous samples, while DNA chips, which can be used to analyze many samples, are of limited practical use due to the high cost of allele-specific fluorescent probes (Campbell et al., 2015). Advances in next-generation sequencing (NGS) have greatly reduced sequencing costs, enabling the analysis of nearly countless SNPs. However, for plants with large genomes, the cost of analyzing many individuals using classical NGS remains prohibitive. Alternative methods have capitalized on reduced genome representation, including genotyping-by-sequencing (GBS) and restriction-site-associated DNA sequencing (RAD-seq), which have been employed in crops to lower genome complexity (Davey et al., 2011; Deschamps et al., 2012; Truong et al., 2012; Narum et al., 2013; Meek and Larson, 2019). However, RADseq is limited to SNPs adjacent to enzyme cutsites while GT-seq methods can target any SNP in the genome, including SNPs associated with phenotypic variation. In addition, GT-seq usually produces more reliable genotyping due to a high read depth than previous methods (Mckinney et al., 2018).

Amplicon sequencing has paved the way for genotyping many samples by sequencing a library of polymerase chain reaction (PCR) products amplified from sets of target genes. Amplicon sequencing technology was applied to an exploration of genome variation across 17 starch biosynthesis genes in 233 rice accessions (Kharabian-Masouleh et al., 2011). Another type of targeted sequencing approach is targeting induced local lesions in genomes (TILLING), which is typically used to identify rare mutations in a small number of genes (Tsai et al., 2011; Thapa et al., 2019). However, both techniques are limited to discovering variants in a few selected genes and are not easily amenable to identifying variants in large samples. Multiplex PCR targeted amplicon sequencing (MTA-seq) addresses these shortfalls by assigning a specific barcode to the amplicons of each sample to facilitate demultiplexing and simplify library preparation through highly multiplexed PCR. However, limitations in barcode options limit sequencing to 96 samples per lane. Using MTA-seq, only small number of samples, including eight parental lines and their F_1_ hybrids of purple false brome (*Brachypodium distachyon*), were used for genotyping (Onda et al., 2018).

Genotyping-in-thousands by sequencing (GT-seq) is based on pooling PCR amplicons with dual barcodes generated by multiplex PCR against multiple target loci and sequencing the resulting library in a single lane. GT-seq is a cost-effective and flexible method to genotype multiple loci of interest by overcoming the limitation of sample size through dual barcoding (Campbell et al., 2015). In the GT-seq pipeline, SNPs are called from sequencing reads, offering the full range of SNPs that map to a given target amplicon. As the cost in GT-seq decreases with increasing pooling, this method is suitable for genotyping many samples at once (Bootsma et al., 2020). The GT-seq method has been widely applied to fish, marine, animal, insect and plant species (Baetscher et al., 2018; Coster et al., 2018; Curran et al., 2018; Förster et al., 2018; Bhattacharyya et al., 2019; Natesh et al., 2019; Yang et al., 2019; Zhao et al., 2019; Batz et al., 2020; Cruz-López et al., 2020; Eriksson et al., 2020; Lukindu et al., 2020; Nakayama et al., 2020; Samuk et al., 2020; Schmidt et al., 2020; Sjodin et al., 2020; Sriboon et al., 2020; Nasti et al., 2021; Srivathsa et al., 2021). In the case of plant, however, there has been no report of optimization GT-seq for thousand samples, and GT-seq was applied for only limited number of samples and mutation detection.

In this study, we demonstrate the applicability of the GT-seq method to pepper samples. We successfully converted previously developed Fluidigm^®^ SNP markers and SNP markers developed through GBS to GT-seq markers. We illustrate the use of the resulting GT-seq markers by conducting various analyses across pepper populations.

## Materials and Methods

### Plant Materials

A total of 1,436 individuals were used, including *Capsicum annuum*, *C. baccatum*, *C. chinense*, *C. frutescens*, and *C. chacoense* accessions. A subset of the pepper core collection (Lee et al., 2016) consisting of 30 *C. annuum* accessions, 26 *C. baccatum* accessions, 25 *C. chinense* accessions, 7 *C. frutescens* accessions, and 1 *C. chacoense* accession was used to test GT-seq markers. F_1_ hybrids were obtained from the crosses *C. annuum* “CM334” × *C. baccatum* ‘PBC81’ (CPIL) and *C. annuum* “Jeju” × *C. chacoense* “PI260433” (PJIL). The CPIL backcross populations and the PJIL F_2_ and backcross populations (with *C. annuum* as a recurrent parent for both populations) were used to develop SNP markers using GBS and test GT-seq markers (Table 1). Plant materials were provided from National Agrobiodiversity Center, Rural Development Administration. We complied guidelines and legislation of National Agrobiodiversity Center, Rural Development Administration.

**TABLE 1:** Summary of plant populations used for GT-seq library construction.

Name	Species	Type	Size
Natural population	*C. annuum*	Accessions	30
	*C. baccatum*	Accessions	26
	*C. chinense*	Accessions	24
	*C. frutescens*	Accessions	7
	*C. chacoense*	Accessions	1
CPIL	*C. annuum* × *C. baccatum*	F_1_	6
		Backcross populations	854
PJIL	*C. annuum* × *C. chacoense*	F_1_	8
		F_2_	192
		Backcross population	188
Seed company	*C. annuum*	Breeding lines	4

### Genomic DNA Extraction

Genomic DNA was extracted from leaf tissue collected in Matrix^TM^ Blank and Alphanumeric Storage tubes by the cetyltrimethylammonium bromide (CTAB) method (Doyle and Doyle, 1987). Extracted DNA was quantified and its concentration adjusted to 15–25 ng/μL using a Take 3 spectrophotometer (BioTek, Winooski, VT, United States).

### Genotyping-by-Sequencing and Single Nucleotide Polymorphism Calling

A GBS library was constructed for 80 individuals from the CPIL BC_1_F_1_ population, the two parental accessions, and their F_1_ hybrids as previously described (Jo et al., 2017). The samples were doubled-digested, ligated, amplified, and quantified separately, and then the amplicons were pooled into one pool for single-lane sequencing. About 400 ng genomic DNA was double-digested with the restriction enzymes *Mse*I and *Eco*RI, before adaptors were ligated to digested DNA fragments with different barcode combinations. Each barcode-ligated fragment was then amplified by PCR with primers encompassing the *Mse*I and *Eco*RI restriction sites. After amplification, the samples were quantified with a Bioanalyzer DNA 1000 chip (Agilent Technologies, Santa Clara, CA, United States). Equal amounts from each sample were pooled for sequencing. Sequencing was performed by Macrogen (Seoul, South Korea) on a HiSeq2000 platform. Raw reads were demultiplexed based on the barcode, before barcode and adapter sequences were trimmed with CLC genomics workbench software version 8.5.1. Demultiplexed reads were then mapped to the “CM334” v1.6 reference genome and “UCD10X” v1.0 reference genome (Kim et al., 2014; Hulse-Kemp et al., 2018) with Burrows-Wheeler Aligner (BWA) version 0.7.12 (Li, 2013) and converted to BAM files. Mapped BAM files were then read, grouped, and sorted using Picard 1.119 samtools 1.1. UnifiedGenotyper version 3.3 from the Genome Analysis ToolKit (GATK) was used for variant calling, while SelectVariants version 3.3 of GATK was used to filter biallelic SNPs with a minimum quality score (Taranto et al., 2016) of 30, SNP as a variant type, minimum sequencing depth of 5, and maximum mismatches of 10.

### Genotyping-in-Thousands by Sequencing Primer Design

**FIGURE 1:**
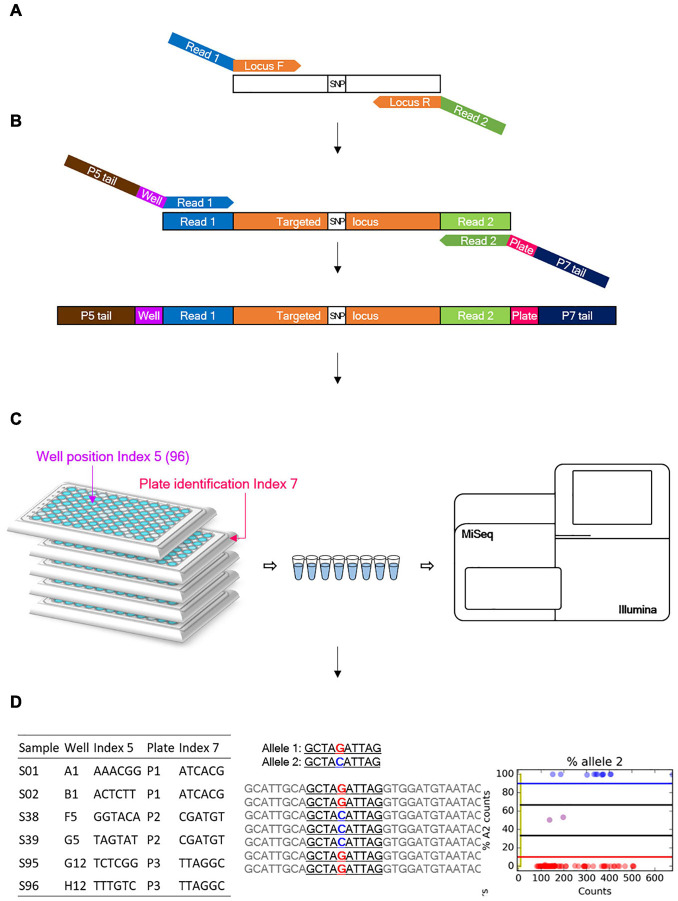
Overview of the genotyping-in-thousands by sequencing (GT-seq) workflow (Campbell et al., 2015). **(A)** First PCR step: multiple target loci are amplified from a panel of multiplexed primers. **(B)** Second PCR step: index barcodes are attached. **(C)** Normalization and sequencing step: all steps are conducted on each plate separately. **(D)** Each library is then quantified and pooled into a single lane for sequencing on a Miseq or Nextseq platform.

Genotyping-in-thousands by sequencing (GT-seq) primers were designed within 100 bp on either side of a given SNP, based on existing studies (Campbell et al., 2015). Primers for previously developed markers for Fluidigm EP1^TM^ (Kang et al., 2014) and newly designed GBS-derived SNP makers were designed by adding Illumina sequencing primer tags, existing custom-read 1 [TCTACACGTTCAGAGTTCTACAGTCCGACGATC] and custom-read 2 [GTGACTGGAGTTCAGACGTGTGCTCTTCC GATCT] sequences (Campbell et al., 2015) for the first PCR step (Figure 1), and locus-specific sequences ranging from 49 to 73 bp in length. Commercially synthesized primers were diluted to a concentration of 100 nM for forward and reverse primers. PrimerPooler v1.41 was used to avoid inter- and intra-primer hybridization and improve the combination of primer pairs.

### Genotyping-in-Thousands by Sequencing Library Preparation

Library preparation was conducted according to the GT-seq library preparation protocol (Campbell et al., 2015), with slight modifications to the number of PCR cycles and the purification volume. In the first step of locus-specific multiplex PCR amplification of target loci, each PCR reaction consisted of 2 μL genomic DNA, 3.5 μL 2 × master mix from the Qiagen Multiplex PCR plus kit, and 1.5 μL pooled primers for 92–288 markers (Figure 1A). PCR amplification was performed with the following conditions: 15 min at 95°C; 5 cycles of 30 s at 95°C, 1.9°C ramp down to 30 s at 57°C and 2 min at 72°C; 15, 20, or 25 cycles of 30 s at 95°C, 30 s at 65°C, and 30 s at 72°C; and 10 min at 4°C. Samples were held in 96-well plates sealed with Thermo Scientific adhesive sealing sheets. The second step for GT-seq library preparation was performed exactly as described previously, with dual barcode indexing, second PCR amplification, and library normalization for each plate (Figures 1B,C). From each normalized sample of each plate, 10 μL was taken out and pooled into a 1.5-mL microtube. To purify the GT-seq library, 500 μL of each pool was mixed with 250 μL AM-PureXP Magnetic beads using a vortex mixer. The mixture was then placed on a magnetic rack until the supernatant cleared, and 750 μL supernatant was transferred to a new 1.5-mL microtube. After addition of 350 μL of beads, the mixture was mixed with a vortex mixer and placed on a magnetic rack until the supernatant cleared. The supernatant was discarded, and the attached beads were washed with 1000 μL 70% ethanol with pipetting onto the beads two or three times. The beads were air-dried and the bound DNA eluted in 50 μL TE buffer, pH 8.0. The purified libraries were transferred to 0.2-mL eight-strip tubes in the same order as the plates. The purified libraries were quantified using a Bioanalyzer DNA Chip (Agilent Technologies, Santa Clara, CA, United States), and the same amount of library was pooled in a single lane for sequencing (Miseq or Nextseq) (Figure 1C).

### Genotyping-in-Thousands by Sequencing Data Analysis

Paired-end sequencing data were trimmed using cutadapt (Martin, 2011) to remove the adapter sequence AGATCGGAAGAGCACACGTCTGAACTCCAGTCAC from read 1 and GATCGTCGGACTGTAGAACTCTGAACGTGTA GA from read 2. Trimmed paired-end reads were then merged into a single fastq file using FLASH2-2.2 except for reads with small overlap sizes below 9 bp (Magoč and Salzberg, 2011). Merged reads were genotyped using GTseq_Genotyper_v3.pl, followed by GTseq_GenoCompile_v3.pl to compile the data into a genotype file. The resulting csv files were summarized using GTseq_SummaryFigures_v3.py to obtain read distribution, ratio of primer reads on-target (reads with forward primer and probe/reads with forward primer), genotyping rate at each locus, and statistics of the library (Campbell et al., 2015) (Figure 1C). These summary files for GT-seq libraries were used to compare genotyping rates per library generated by different methods. Genotyping frequencies were compared between compiled csv files and Fluidigm genotype data.

### Visualization of Genotyping-in-Thousands by Sequencing Marker Distribution

Distribution of GT-seq markers, converted from Fluidigm markers and GBS SNPs, was drawn with MapChart 2.30 software using the physical position of all markers according to the “CM334” v1.6 genome.

## Results

### Marker Assessment for Genotyping-in-Thousands by Sequencing Primer Conversion

**FIGURE 2:**
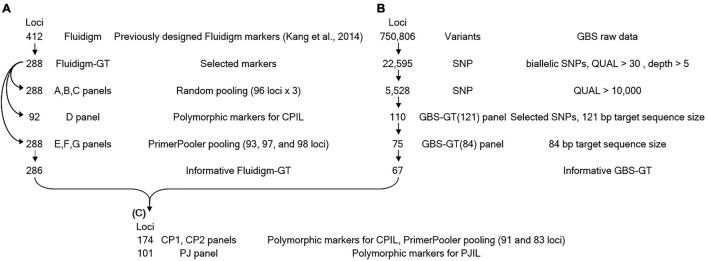
Strategy for developing GT-seq panels. **(A)** The panels of Fluidigm-GT-seq primer converted from Fluidigm markers. **(B)** The panels of GBS-GT-seq primer designed from GBS-SNP. **(C)** The panels by mixing the Fluidigm-GT-seq and GBS-GT-seq primers.

We converted SNP markers previously developed for the Fluidigm genotyping platform to GT-seq markers. Of all Fluidigm SNP markers, we selected 288 based on their distribution along the pepper chromosomes, represented in at least 7 individual accessions out of a set of 677 which were a part of 3,821 *Capsicum* accessions (Lee et al., 2016), with a minor allele frequency of at least 0.005 and an average of 0.076 (Figure 2 and Supplementary Table 1). We converted the corresponding 288 Fluidigm primer pairs to Fluidigm-GT-seq primers by adding sequencing tags. The Fluidigm-GT-seq primers were 49–73 bp in length including the tag sequence, with an average target size of 78 bp. excluding the tag sequence.

To test the efficacy of these SNP markers to genotype a genomic segment introgressed to *C. annuum* from *C. baccatum*, we genotyped a 80 backcross population derived from a cross between (*C. annuum* “CM334” × *C. baccatum* “PBC81”) and “CM334,” designated CPIL BC_1_F_1_, by GBS (Supplementary Table 2). We called 750,806 variants from the GBS data, from which we selected 22,595 SNPs after excluding multiallelic SNPs and SNPs with a phred-scaled quality score for the assertion made in alternative genotype call (Taranto et al., 2016) below 30, a coverage depth below 5, and more than 10 missing call. Of these SNPs, 5,528 had a QUAL > 10,000 and harbored only the homozygous reference allele and alternative allele for each parent, as expected for a polymorphic region between “CM334” and “PBC81.” We then extracted their corresponding 121-bp target sequences and used them as query for a Basic Local Alignment Search Tool (BLAST) analysis against the reference pepper genome. We finally selected 110 SNPs distributed equally across chromosomes from the set of sequences with BLAST hits below 180 (Supplementary Table 3). To amplify the polymorphic sequences, we then designed 75 pairs of GBS-GT-seq primers with a target sequence size of 84 bp and another set of 110 pairs of primers with a target sequence size of 121 bp (Figure 2 and Supplementary Tables 2–4).

To assess the genome coverage of GT-seq markers, we compared the genotypes of GT-seq markers and GBS-SNPs, which are flanked with GT-seq markers. The total genotype matching rate was 92.5% for the polymorphic markers, where the matching rates of Fluidigm-GT-seq markers and GBS-GT-seq markers with GBS-SNPs were 91% and 93.7%, respectively (Supplementary Table 2A). The average of recombination points was estimated to be 7.7 (Supplementary Table 2C), and the average number of polymorphic markers in each chromosome was 20.2, demonstrating that polymorphic GT-seq markers exceed recombination breakpoints and could cover the genomic segments of *C. baccatum* in CPIL BC_1_F_1_ population.

### Optimizing Multiplex PCR: Number of PCR Cycles

**FIGURE 3:**
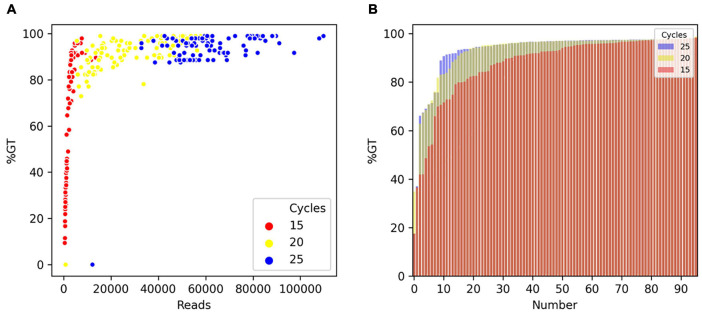
Comparison of genotyping rate as a function of the number of PCR cycles. **(A)** Scatterplot of genotyping rate (%GT) as a function of read number after 15 (red), 20 (yellow), or 25 (blue) PCR cycles. **(B)** Genotyping rate of loci in the panel. Number of PCR cycles: 15 (red), 20 (yellow), or 25 (blue).

To optimize the number of PCR cycles for locus-specific multiplex PCR, we tested the effect of varying the number of PCR cycles using a randomly selected set of 96 Fluidigm-GT-seq primers (panel A) out of the 288 Fluidigm-GT-seq primers mentioned above to genotype 96 selected accessions from the pepper core collection (named natural population). Accordingly, we performed PCR amplification for the preparation of the GT-seq library with 15, 20, or 25 cycles (Figure 1A). We sequenced the resulting PCR amplicons as paired-end reads, resulting in a total of 18.9 M GT-seq reads. After demultiplexing and merging of both paired-end reads, we obtained 0.25, 2.7, and 6.0 M reads from PCR amplification with 15, 20, and 25 cycles, respectively, demonstrating that the number of reads sequenced increases with the number of PCR cycles. More reads contributed to a higher genotyping rate, reaching 53%, 91%, and 94% from amplification for 15, 20, and 25 cycles, respectively. Of the 96 markers, the number of markers exceeding the 90% genotyping rate across the 96 samples rose with the number of PCR cycles, from 42 with 15 cycles to 78 with 20 cycles and 87 with 25 cycles. In summary, the increased genotyping rate at each marker were obtained with the number of PCR cycles, with the highest genotyping rate obtained with 25 PCR cycles (Table 2 and Figure 3).

**TABLE 2:** Comparison of genotyping rates with different numbers of PCR cycles for GT-seq library construction.

PCR cycles of first amplification	15	20	25
Samples in library	96	96	96
Samples over 90%GT^a^	14	62	77
Multiplexed loci in panel	96	96	96
Loci over 90%GT	42	78	87
Raw reads (million)	0.3 M	2.8 M	6.1 M
Average on-target reads (%^b^)	70.1	89.1	91.4
Average genotyping (%^c^)	52.9	91.4	94.4

*^a^Genotyping rate.*

*^b^Percentage of reads containing locus-specific forward sequence in the total reads.*

*^c^Percentage of genotyped samples.*

### Optimizing Multiplex PCR: Number of Primers

We next investigated the number of primer pairs that can be included in a multiplex PCR primer panel during preparation of a GT-seq library. From the 288 Fluidigm-GT-seq markers, we excluded the 96 markers used above to optimize the number of PCR cycles and arbitrarily divided the remaining 192 Fluidigm-GT-seq primers into two panels (panels B and C). Separately, we selected 92 Fluidigm-GT-seq primers out of the full set of 288 primers that showed polymorphisms between the parental lines of the CPIL population as another panel (panel D). We also combined the primer sets included in panels A, B, and C to generate panels AB, BC, and AC, with 192 Fluidigm-GT-seq primers. We then used seven panels (A, B, C, D, AB, BC, and AC) to prepare libraries from 48 samples using 25 PCR cycles (Table 3).

**TABLE 3:** Summary of GT-seq results in seven panels containing different numbers of multiplexed primers.

Panel	A	B	C	D	AB	BC	AC
Samples in library	96	48	48	48	48	48	48
Multiplexed loci in panel	96	96	96	92	192	192	192
Average size of amplicon (bp)	77	79	79	79	78	79	78
Raw reads	1,771,502	889,902	707,398	984,394	659,869	640,234	956,879
Read depth	192	193	154	214	72	69	104
Samples over 90%GT	40	23	19	18	10	1	15
Average on-target reads (%)	44.8	42.7	39.9	25.7	36.6	25.9	35.4
Average genotyping (%)	87.0	84.9	88.2	83.4	71.3	63.7	81.15

*Read depth = Reads/(Loci × Samples).*

We sequenced the resulting libraries on one Miseq lane, generating 13.7 M reads. After demultiplexing, the libraries derived from panels A, B, C, and D counted about 4.4 M reads each, with an average read depth of 189 at each SNP. The remaining three libraries (AB, BC, and AC) resulted in 2.3 M reads, with an average read depth of 82. The average genotyping rate was 86.1% for panels A, B, C, and D and 72.2% for panels AB, BC, and AC (Table 3). In summary, the genotyping rate decreased with the number of primers in a single panel, with the lowest genotyping rate obtained with 192 primers in a single panel. Thus, we demonstrated the optimum number of primers is around 96.

### Optimizing Multiplex PCR: Primer Combinations

To minimize interference between primers during multiplex PCR, we selected pools of primer pairs from 288 Fluidigm-GT-seq primers, consisting of approximately 96 primer pairs, with PrimerPooler, resulting in pools of 93, 97, and 98 primer pairs (panels E, F, and G, respectively) with the lowest predicted interference between primers. We then prepared and sequenced libraries with primer panels E–G for 384 samples. We thus obtained 9.6 M reads, of which 3.5, 2.8, and 3.4 M originated from the libraries derived from panels E, F, and G, respectively. The genotyping rates were 79.2% (panel E), 79.3% (panel F), and 88.7% (panel G), with an average of 82.4% (Table 4). We then compared the genotyping rates of 48 BC_1_F_1_ plants genotyped at 59 commonly utilized loci with pools of randomly selected primer combinations (A, B, and C) and primer combinations selected by PrimerPooler (E, F, and G). The mean genotyping rate was 85% for panels A–C and 89% for panels E–G (Supplementary Figure 1 and Supplementary Table 5). These results demonstrated that genotyping rate can be improved by minimizing inter-primer interference within primer panels.

**TABLE 4:** Summary of GT-seq results in four panels containing different combinations of multiplexed primers using PrimerPooler.

Panel	E^b^	F^c^	G^d^	GBS-GT
Samples in library ^a^	384	384	384	384
Multiplexed loci	93	97	98	110
Marker type	Fluidigm-GT	Fluidigm-GT	Fluidigm-GT	GBS-GT
Number of genotypes	35,712	37,248	37,632	42,240
Raw reads	3,449,723	2,792,330	3,448,327	4,410,556
Read depth	97	75	72	104
Average size of amplicon (bp)	78	77	79	121
Average on-target reads (%)	70.2	79.4	81.7	40.6
Average genotyping (%)	79.2	79.3	88.7	84.3
Samples over 90%	99	56	230	130

*^a^80 BC_1_F_1_ and 269 BC_2_F_1_ population, 12 parents, and 23 F_1_ hybrids. Sixteen library plates were sequenced in one lane.*

*^bcd^Divided panels from 288 sets of converted targeted sequencing primer pairs using PrimerPooler.*

### Compatibility of Fluidigm-Genotyping-in-Thousands by Sequencing and Genotyping-by-Sequencing-Genotyping-in-Thousands by Sequencing Markers for Multiplexing

To investigate the compatibility of Fluidigm-GT-seq markers which were converted from Fluidigm primers designed by Fluidigm D3^TM^ Assay Design and GBS-GT-seq markers which were designed from GBS-SNP, we determined the genotyping rates when mixing two markers. From a list of SNPs obtained by GBS, we designed 110 GBS-GT-seq markers with a target sequence size of 121 bp. We then prepared two libraries: one using 96 GBS-GT-seq markers and another using 14 GBS-GT-seq markers and 77 Fluidigm-GT-seq markers (78 bp in length) before sequencing by GT-seq. We obtained a genotyping rate of 8.8% in the first library based solely on GBS-GT-seq markers, and only 11 GBS-GT-seq markers were genotyped, indicating that 121-bp GBS-GT-seq markers have primer interference (Supplementary Table 6A). In contrast, the second library prepared by mixing the two types of markers yielded genotyping rates of 81.6% for Fluidigm-GT-seq markers, while none of the GBS-GT-seq markers returned useful information, indicating that 121-bp GBS-GT-seq markers and 78-bp Fluidigm-GT-seq markers cannot be mixed for GT-seq library preparation (Supplementary Table 6B). To attempt to fix these issues, we divided the 110 GBS-GT-seq primer pairs into 30, 40, and 40 pairs [panel GBS-GT(121)] to minimize interference. To demonstrate a single second PCR for library construction, each amplicon of the multiplex PCR using three pairs [panel GBS-GT(121)] was pooled by each sample (Table 4). The genotyping rate was 84.3% for the library of the GBS-GT(121) panel (Table 4). We reduced the target sequence size of GBS-GT-seq markers to be closer to that of Fluidigm-GT-seq markers. We designed 75 new GBS-GT-seq markers, named GBS-GT(84) panel, with an average target sequence size of 84 bp from the initial set of 110 GBS-GT(121) panel (Figure 1 and Supplementary Table 4). We then mixed the GBS-GT-seq(84) primer pairs with Fluidigm-GT-seq primer pairs for multiplexing and applied the pools of primers to genotyping the CPIL and PJIL populations (Figure 1). As above, we divided the two sets of primer pairs into two panels (CP1 and CP2) to genotype the CPIL population, while we mixed 30 GBS-GT-seq(84) primer pairs and Fluidigm-GT-seq primer pairs into one panel (PJ) to genotype the PJIL population, using PrimerPooler, on one Nextseq lane (Figure 1 and Table 5). Using this approach and final optimization of the number of PCR cycles, the number of primers, primer combinations, and sequencing platform, we reached average genotyping rates of 96.92% and 94.85% for the CPIL and PJIL populations, respectively (Table 5). In summary, we discovered that the target sequence size for GT-seq markers must be in the same range to perform well during multiplex PCR and the single panel size 101 (PJ) is optimum size in the first multiplex amplification. To avoid primer interference, the different panel should be amplified separately in the first amplification (CP1 and CP2). Then the first amplicon could be combined before second amplification to increase the panel size.

**TABLE 5:** Summary of GT-seq results when mixing GBS-GT-seq markers and Fluidigm-GT-seq markers in the Nextseq platform.

Library	CPIL		PJIL
Samples in library	480		384
Panel	CP1	CP2	PJ
Total number of multiplexed loci	91	83	101
Fluidigm-GT-seq markers	57	50	71
GBS-GT-seq markers	34	33	30
Number of genotypes	83,520		38,784
Raw reads	115,282,386		56,960,087
Read depth	1,380		1,469
Average on-target reads (%)	62.39		54.6
Average genotyping (%)	96.92		94.85
Samples over 90%GT	470		347

### Genotyping Rates Using Different Sequencing Platforms

To increase the number of samples analyzed, we tested two platforms with different read output per lane: the Illumina Miseq platform can produce up to 30 M reads, but Illumina Nextseq can reach up to 260 M reads per lane. We thus compared the results of sequencing for 384 samples on Miseq and 864 samples on Nextseq (Tables 4, 5). The two platforms produced 14.1 M (Miseq) and 172.2 M (Nextseq) raw reads, resulting in 152,832 and 122,304 genotypes (Supplementary Table 7), with read depths of 92 (Miseq) and 1,408 (Nextseq), representing a difference of more than 15-fold. We obtained average genotyping rates of 82.8% from Miseq sequencing and 96.6% from Nextseq sequencing (Supplementary Table 7). In conclusion, we observed a higher genotyping rate at all markers with greater sequencing output. Therefore, the Nextseq sequencing platform is suitable for genotyping many samples with high accuracy.

### Comparison of Fluidigm Single Nucleotide Polymorphism and Genotyping-in-Thousands by Sequencing Genotyping

**FIGURE 4:**
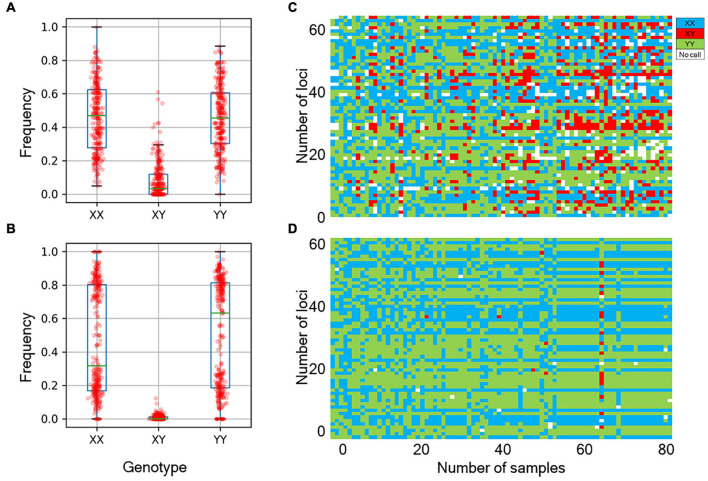
Comparison of genotypes between the Fluidigm system and GT-seq. **(A)** Distribution of genotype frequencies at 288 loci, as determined by the Fluidigm system. **(B)** Distribution of genotype frequencies at 288 loci by GT-seq. **(C)** Distribution of fluorescent genotype makers by Fluidigm. **(D)** Distribution of GT-seq genotypes that were the same individuals at the same markers.

**FIGURE 5:**
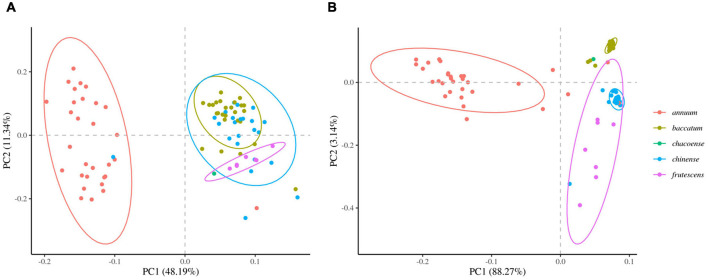
Principal component analysis (PCA) of population structures in natural populations. **(A)** PCA of genotypes derived from Fluidigm. **(B)** PCA of genotypes obtained from GT-seq.

We next determined the accuracy of genotyping by Fluidigm-GT-seq. Accordingly, we used 288 Fluidigm markers and 288 converted Fluidigm-GT-seq markers to genotype and assess heterozygosity of 88 accessions commonly used with the two genotyping methods. Fluidigm markers revealed an average heterozygosity ranging from 0 to 48.0%, and the mean value was 16.1% in the tested accessions. Fluidigm-GT-seq markers produced more binary results, with an average heterozygosity ranging from 0 to 22.5%, and the mean value was 0.8% for 282 markers, with six markers returning no useful information (Figures 4A,B and Supplementary Table 8A). The only four accessions exceeding 2% heterozygosity, across the 288 Fluidigm-GT-seq markers, of the 88 accessions were *C. annuum* “RS205,” “RS203,” “CM334,” and “VK-515S” with 22.5%, 20.6%, 7.9%, and 2.2% heterozygosity, respectively (Supplementary Table 8A). In contrasts, 59 accessions exceeded 2% heterozygosity across the 288 Fluidigm markers (Supplementary Table 8A). We also investigated the distribution of 60 markers commonly used in Fluidigm and Fluidigm-GT-seq across 80 accessions (Figures 4C,D). Genotype information appeared much clearer with Fluidigm-GT-seq markers, suggesting that the frequent heterozygosity observed with Fluidigm markers might reflect genotyping failure. To determine if the genotyping information generated with Fluidigm and Fluidigm-GT-seq markers clustered accessions according to their *Capsicum* species, we performed a principal component analysis (PCA). The first two components, PC1 and PC2, explained up to 59.5% of variance when using Fluidigm-based data, while Fluidigm-GT-seq genotypes increased the explained variance to 91.4% (Figure 5). In addition, PCA of genotyping data generated by Fluidigm-GT-seq largely clustered accessions based on their species, with a few exceptions.

### Distribution of Markers

Finally, we determined the genomic coordinates of all 288 Fluidigm-GT-seq markers using the pepper reference genome CM334 v1.6 and displayed their genomic locations along the genome. We performed the same analysis with 110 GBS-GT-seq markers (Supplementary Figure 2).

We next exploited these GT-seq markers to genotype a natural population: from 288 Fluidigm-GT-seq markers, 286 were informative, while 77 of 110 GBS-GT-seq markers (121 bp in size) were informative across the population. Similarly, 67 of 75 GBS-GT-seq(84) markers were polymorphic across the population. Finally, we selected 256 markers from 286 Fluidigm-GT-seq markers with a genotyping rate of at least 50% as useful markers (Supplementary Figure 2 and Supplementary Table 2).

## Discussion

In this study, we demonstrate that GT-seq can be applied to plants, in this case using various chili pepper species. We converted Fluidigm markers developed for chili peppers to GT-seq markers, as well as SNP markers identified by GBS, for a total of 332 GT-seq markers. We then established conditions for library preparation with these markers to reach high genotyping rates on either one of two sequencing platforms: Miseq or Nextseq. Reliable genotyping data in diploid plants requires a sequencing depth of at least 30 reads (Gemenet et al., 2020). Based on our sequencing coverage from pooled samples, we estimate that 1,500 samples can be analyzed in one Miseq lane and 13,000 samples in one Nextseq lane for 96 target loci, each represented by one GT-seq marker.

Efficient multiplex PCR is critical during the GT-seq protocol to generate sequencing-compatible amplicons for each sample. A major factor interfering with multiplex PCR is interaction between primers; reducing these interactions is important for optimizing multiplex PCR (Wei et al., 2008). With this in mind, we employed a master mix with a Taq polymerase exhibiting hot-start activity, together with a multiplex PCR plus kit to minimize primer interactions during PCR. Unlike genomic DNA extracted from animals, plant genomic DNA tends to be contaminated with polysaccharides, polyphenols, pectin, and xylan, which may all interfere with multiplex PCR (Wei et al., 2008; Schrader et al., 2012). We therefore increased the number of PCR cycles from 15, which was reported condition for GT-seq. In the case of plant, however, there has been no report of optimization GT-seq for thousand samples, and GT-seq was applied for only limited number of samples and mutation detection in Salmon [7], to 25 during the first multiplex PCR to compensate for reduced PCR efficiency and generate enough amplicons for sequencing. In addition, we used the PrimerPooler tool to minimize primer dimers.

We were surprised to discover that only Fluidigm-GT-seq markers generated useful information when their primer pairs were mixed with those of our newly developed GBS-GT-seq markers during multiplex PCR. We hypothesized that the failure of GBS-GT-seq markers resided in their different size: Fluidigm-GT-seq markers had an average target sequence size of 78 bp, whereas the average target sequence size from GBS-GT-seq markers was 121 bp, possibly preventing amplification during multiplex PCR. Indeed, GBS-GT-seq markers performed as well as Fluidigm-GT-seq markers after a redesign step that brought their average size closer to that of Fluidigm-GT-seq markers.

The average genotyping rates for frequently used markers were 92.6% and 83.1% for Fluidigm markers and Fluidigm-GT-seq markers, respectively (Supplementary Table 8B). However, we observed congruent genotypes from the two sets of markers only for 56.2% of the same markers, with a range per marker from 6.8 to 86% (Supplementary Table 8B). This low matching rate is due to the high ratio of heterozygous calls when using Fluidigm markers, which was 16.1% mean heterozygosity from the natural population, in contrast to the 0.8% mean heterozygosity determined on the same germplasm with GT-seq markers (Supplementary Table 8A). Considering that pepper is self-pollinated, the genotyping results from GT-seq markers are more intuitive and likely more reliable. Another possible explanation is that there are different technologies underlying each genotyping method. Fluidigm markers use fluorescence-based technology, raising the possibility of off-target effects due to the complexity of the pepper genome. By contrast, GT-seq is based on sequencing, which is less prone to false positives. We also performed a PCA using genotyping data generated with Fluidigm or GT-seq markers on a panel of 88 *Capsicum* accessions, which revealed the superiority of GT-seq genotyping.

Conventional genotyping methods such as those relying on fluorescence or chips have a linear increase in cost with higher sample numbers. With classic sequencing technologies, the sequencing coverage is limited to genotyping multiple polymorphic loci, but it is difficult to adjust the numbers of polymorphic sites and samples that can be analyzed. Our method allowed the successful genotyping of over 1,000 samples on a single sequencing lane at several hundred polymorphic sites distributed throughout the pepper genome. With the optimum panel size of 100 loci for multiplex PCR, we combined the first amplicon panels (CP1 and CP2) for the second PCR to increase the panel size of the final library. In this way, it is expected to increase the panel size up to 50–500 loci by adding up the first panels.

Fast genotyping analysis is essential in molecular breeding. There are various Illumina-based sequencing platforms whose read output and runtime vary. Early GT-seq studies performed sequencing on a HiSeq1500 with the estimated sequencing depth is over 3,000 (192 SNPs and 2068 samples) and the genotyping rate is 96.4% (Campbell et al., 2015). Later GT-seq experiments switched to Miseq, a benchtop sequencer (Bootsma et al., 2020). In this work, we used Nextseq and Miseq for a direct comparison. Nextseq generated 15 times greater read depth than Miseq, resulting in a higher genotyping rate with the sequencing depth of 1,408 and the genotyping rate of 96.6% (Supplementary Table 7). Despite the optimum panel size is smaller than the original GT-seq, we achieved a slightly increased genotyping rate with half of the previously genotyping rate [7]. Therefore, we propose that Nextseq combined with GT-seq paves the way to analyzing more samples, thereby reducing the cost per sample. Assuming a read depth of 100 for 96 target loci, the maximum number of samples amenable to analysis via the Miseq and Nextseq platforms would be 1,500 and 13,000, respectively. Thus, the sequencing cost per sample drops from $1.32 using Miseq to $0.14 using Nextseq. Additionally, we demonstrated normalization of the GT-seq library using Agencourt AMPure XP Bead as an alternative approach. This method provides the potential to reduce the normalization price.

In this work, we developed a set of GT-seq markers to genotype peppers. This study is the first case of applying GT-seq to over thousand samples in a crop plant. When conducting crop breeding and population genetics, hundreds or thousands of plants are analyzed. GT-seq, with its low cost per sample, could be used in multiple plants and crops.

## Data Availability Statement

The original contributions presented in the study are publicly available. This data can be found here: National Center for Biotechnology Information (NCBI) BioProject database under accession number PRJNA761284.

## Author Contributions

B-CK, JJ, and YK conceived and designed the experiments. JJ and YK performed experiments. JJ, YK, and GK analyzed the data. JJ and B-CK drafted the manuscript. JJ, B-CK, and J-KK revised the manuscript. All authors contributed to the article and approved the submitted version.

## Conflict of Interest

The authors declare that the research was conducted in the absence of any commercial or financial relationships that could be construed as a potential conflict of interest.

## Publisher’s Note

All claims expressed in this article are solely those of the authors and do not necessarily represent those of their affiliated organizations, or those of the publisher, the editors and the reviewers. Any product that may be evaluated in this article, or claim that may be made by its manufacturer, is not guaranteed or endorsed by the publisher.
