# Supplementary material for: Development of a Panel of Genotyping-in-Thousands by Sequencing in Capsicum
Source: Front Plant Sci. 2021 Oct 26;12:769473. doi: 10.3389/fpls.2021.769473 (PMC8576353; doi:10.3389/fpls.2021.769473)
Supplement: Supplementary file 1 [file Data_Sheet_1.docx]

**
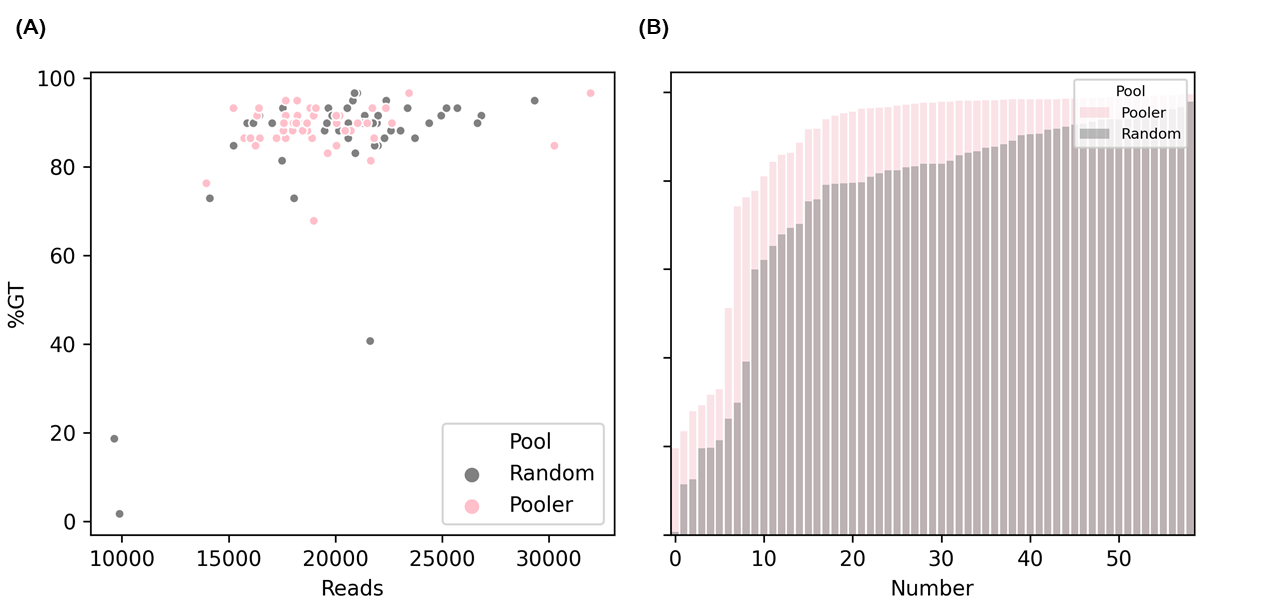
**

Supplementary Figure S1. Comparison of GT-seq results as a function of primer pooling. **(A)** Genotyping rates of each sample. **(B)** Genotyping rates of loci in the reads. Gray and pink indicate random pooling and PrimerPooler pooling, respectively.
