# Supplementary material for: Development of a Panel of Genotyping-in-Thousands by Sequencing in Capsicum
Source: Front Plant Sci. 2021 Oct 26;12:769473. doi: 10.3389/fpls.2021.769473 (PMC8576353; doi:10.3389/fpls.2021.769473)
Supplement: Supplementary file 2 [file Data_Sheet_2.docx]

Supplementary Figure S2. Physical positions of 355 GT-seq markers anchoring to chromosomes of the CM334 v1.6 genome, out of the 398 GT-seq primers, consisting 245 Fluidigm-GT-seq primers and 110 GBS-GT-seq primers. Left rulers indicate physical distance. Blue indicates 218 Fluidigm-GT-seq markers anchoring to chromosomes out of the selected 256 useful markers. Green indicates location of 75 size-converted GBS-GT-seq primers. Yellow indicates noninformative marker out of two noninformative Fluidigm-GT-seq markers.
