# Supplementary material for: Development of a Panel of Genotyping-in-Thousands by Sequencing in Capsicum
Source: Front Plant Sci. 2021 Oct 26;12:769473. doi: 10.3389/fpls.2021.769473 (PMC8576353; doi:10.3389/fpls.2021.769473)
Supplement: Supplementary file 7 [file Table_5.docx]

Supplementary Table S5. Comparison of GT-seq results as a function of primer pooling.

| Multiplexed panel | Random pooling | PrimerPooler |
| --- | --- | --- |
| Samples in library ^a^ | 48 | 48 |
| Average on-target reads (%) | 36 | 24 |
| Average genotyping (%) | 85 | 89 |
| Samples over average | 38 | 27 |
| Samples over 90% | 18 | 17 |

^a^ 48 BC_1_F_1_ were selected from 384 samples to compare efficiency between random pooling and PrimerPooler.
