# Supplementary material for: Development of a Panel of Genotyping-in-Thousands by Sequencing in Capsicum
Source: Front Plant Sci. 2021 Oct 26;12:769473. doi: 10.3389/fpls.2021.769473 (PMC8576353; doi:10.3389/fpls.2021.769473)
Supplement: Supplementary file 9 [file Table_7.docx]

Supplementary Table S7. Comparison of different sequencing platforms tested for GT-seq.

|  | Miseq | Nextseq |
| --- | --- | --- |
| Cycle | 300 | 150 |
| Cost | $1,980 | $1,880 |
| Run time | 24 hours | 15 hours |
| Read length | 2 × 150 bp | 2 × 75 bp |
| Raw reads | 14.1 M | 172.2 M |
| Number of genotypes | 152,832 | 122,304 |
| Read depth | 92 | 1,408 |
| Cost per genotype | $1.32 | $0.15 |
| Average GT rate | 82.8% | 96.6% |
